# Effects of hyperbaric oxygen therapy on the expression levels of the inflammatory factors interleukin-12p40, macrophage inflammatory protein-1β, platelet-derived growth factor-BB, and interleukin-1 receptor antagonist in keloids

**DOI:** 10.1097/MD.0000000000019857

**Published:** 2020-04-17

**Authors:** Yan Hao, Xinhang Dong, Mingzi Zhang, Hao Liu, Lin Zhu, Youbin Wang

**Affiliations:** a Department of Plastic Surgery, Peking Union Medical College Hospital; b Plastic Surgery Hospital, Chinese Academy of Medical Sciences & Peking Union Medical College (CAMS & PUMC), Beijing, China.

**Keywords:** hyperbaric oxygen therapy, inflammatory factors, keloid

## Abstract

**Background:**

: Our study aimed to screen and explore the expression of inflammatory factors in keloid patients and to investigate how hyperbaric oxygen (HBO) therapy affects the expression levels of interleukin-12p40 (IL-12p40), macrophage inflammatory protein-1β (MIP-1β), platelet-derived growth factor-BB (PDGF-BB), and interleukin-1 receptor antagonist (IL-1Ra).

**Objective:**

: 30 patients were randomly selected and divided into the following 3 groups: keloid samples from keloid patients treated with HBO therapy (A), keloid samples from keloid patients treated without HBO therapy (B), and normal control skin samples derived from individuals who had no clear scarring (C). Each group included 10 samples.

**Methods:**

: Inflammatory factors in the keloid tissues were measured with the MILLIPLEX multiplexed Luminex system. Hematoxylin and eosin staining, immunohistochemical staining, and Western blotting were used to observe the morphological differences in different tissues and the expression levels.

**Results:**

: The expression levels of inflammatory mediators, including IL-12p40, MIP-1β, PDGF-BB, and IL-1Ra, in keloid tissues were significantly different from those in samples of normal skin. Hematoxylin and eosin staining showed significantly greater inflammatory infiltration in keloid tissue. Significantly different expression levels were observed in group A, B, and C.

**Conclusion:**

: Significantly altered levels of inflammatory factors in the samples from keloid patients were observed, suggesting that formation of a keloid is potentially related to inflammatory responses. HBO therapy could significantly affect the expression levels of IL-12p40, MIP-1β, PDGF-BB, and IL-1Ra, indicating that the effects of HBO therapy are associated with the attenuation of inflammatory responses.

## 1 Introduction

A keloid is defined as a proliferative skin disease that extends beyond the initial edges of the wound and easily invades the surrounding normal tissues, causing severe dysfunction and shape defects in patients. Many factors, such as aberrant tissue reconstruction, excessive inflammatory responses, and an abnormal immune system, which are involved in the wound healing process, play important roles in the formation and evolution of a keloid, but the mechanism of keloid formation is still unclear.

It is generally acknowledged that a keloid is characterized by a high proportion of overactive fibroblasts, leading to excessive deposition of collagen in the extracellular matrix.^[1]^ However, recent studies have shown that keloid tissue has a high concentration of inflammatory cell aggregation and excessive inflammatory factor expression.^[2]^ It has been demonstrated that inflammatory factors are potentially associated with the formation of keloids. In the study by Zhang, the protein and mRNA expression levels of chemokine-like factor-1, interleukin (IL)-6, IL-8, IL-18, and transforming growth factor (TGF)-β were higher in samples from keloid patients, compared with normal skin tissues and scar tissue.^[3]^ Using the gene chip technology in combination, Chen et al found that some inflammatory factor-related gene expression was significantly up-regulated in keloid fibroblasts.^[4]^

Hyperbaric oxygen (HBO) therapy refers to breathing 100% oxygen before surgery while under increased atmospheric pressure, and it has been widely used in clinical practice, especially in plastic surgery. Previous studies have shown that HBO therapy can achieve satisfactory results in skin grafts and chronic nonhealing wounds.^[5,6]^ Our study measured and assessed the expression levels of inflammatory factors in keloids (with and without HBO therapy) and normal skin tissue, and it aimed to determine whether HBO therapy can reduce the level of inflammatory responses in keloids.

## 2 Methods

### 2.1 Patients, grouping, and sample treatment

From February 2017 to February 2018, 30 patients (15 males and 15 females) with (age: 21–52 years) were selected from the Department of Plastic Surgery, Peking Union Medical College Hospital, and randomly assigned into the following 3 groups: 10 keloid samples from keloid patients (5 males and 5 females) treated with HBO therapy (A), 10 keloid samples from keloid patients (5 males and 5 females) treated without HBO therapy (B), and 10 normal skin samples from patients (5 males and 5 females) without obvious scarring (C). All samples were selected and assigned by the method of random number table, ensuring the randomness of sampling. Average ages by group were: 37.82 ± 7.51 years (A group), 36.71 ± 7.85 years (B group), and 37.39 ± 8.21 years (C group). Age did not differ among these 3 groups (*P* > .05). All tissues were obtained from the chest. The size of the selected keloid tissues is 3 cm∗3 cm to 5 cm∗5  cm. The samples were taken from the centers of the keloid tissues, and the size of all samples is 1 cm∗1 cm. Patients in the A and B groups did not have any rupture and infection on the surface of the keloid. None of the patients were treated with laser, cryotherapy, and topical or local injection. None of the 3 groups had immune-mediated, infectious, and other basic diseases. The diagnosis of a keloid was made and confirmed by a plastic surgeon and pathological examination. The Bioethical Committee of Peking Union Medical College Hospital approved this study. All study participants signed the informed consent form.

Each specimen was divided into the following 3 parts: one part of the specimen was collected for multiplex cytokine immunoassay, one part of the specimen was fixed using 10% formalin solution for paraffin embedding to perform hematoxylin and eosin (H&E) staining and immunohistochemical studies, and the remaining was stored in liquid nitrogen for western blot analysis.

### 2.2 HBO Therapy

Before plastic surgery, patients of the A group underwent HBO therapy for 7 days (once a day, 7 times in total) in an air-pressurized medical hyperbaric chamber with 3 locks and 7 doors. Pressure was increased to 0.2 MPa (2 ATA) at a constant speed within 30 minutes. Then, patients inhaled 100% oxygen through face masks for 60 minutes. The patients were scheduled to undergo surgery 24 hours after the last HBO therapy.

### 2.3 Cytokine measurement

Forty-one cytokines were assessed via the MILLIPLEX MAP system, which is a bead-based multiplexed enzyme-linked immunosorbent assay-like assay. Manufacturer's instructions were adhered to. A 41-plex kit was used to assess levels of the following cytokines: soluble CD40 ligand, Epidermal Growth Factor (EGF), Eotaxin/CCL11, Fibroblast Growth Factor-2 (FGF-2), FMS-like tyrosine kinase-3 (Flt-3) ligand, Fractalkine, granulocyte-colony stimulating factor (G-CSF), granulocyte-macrophage colony-stimulating factor, Growth Related Oncogene (GRO), interferon (IFN)-α2, IFN-γ, IL-1α, IL-1β, interleukin-1 receptor antagonist (IL-1Ra), IL-2, IL-3, IL-4, IL-5, IL-6, IL-7, IL-8, IL-9, IL-10, interleukin-12 (IL-12)p40, IL-12p70, IL-13, IL-15, IL-17, IP-10, monotype chemoattractant protein (MCP)-1, MCP-3, Macrophage Derived Chemokine (MDC) (CCL22), macrophage inflammatory protein-1 (MIP-1)α, MIP-1β, platelet-derived growth factor (PDGF)-AA, PDGF-BB, Regulate on Activation, Normal T cell Expressed and Secreted (RANTES), TGF-α, tumor necrosis factor (TNF)-α, TNF-β, and vascular endothelial growth factor.

### 2.4 H&E staining

All slides were dewaxed in a slide drier and then washed in xylene. After being rehydrated in an ethanol gradient, the slides were stained with hematoxylin and differentiated. Then, slides were counterstained with eosin. Next, the slides were dehydrated with an alcohol gradient (backward concentration) and xylene and mounted.

### 2.5 Immunohistochemical staining

Sections had paraffin removed and were rehydrated, followed by a disruption of endogenous catalase activity with a 3% H_2_O_2_ solution. Slides were then heated for 15 minutes to 95°C in a citrate-based buffer. Blocking was performed at 37°C using goat serum, followed by primary antibody incubation at 37°C in a humid chamber for 2 hours. Primary antibodies were: anti-IL-12p40 (1:200, Abcam, Cambridge, UK), anti-MIP-1β (1:200, Abcam), anti-PDGF-BB (1:200, Abcam), and anti-IL-1Ra (1:200, Abcam). Secondary antibody conjugated to horseradish peroxidase was then used (ZSGB-BIO, Beijing, China), and after a phosphate buffer saline was 3, 30-diaminobenzidine and then hematoxylin were used to stain the slides. Antibody binding was colored brown under the light microscope, with images captured using a digital camera. The positive condition was measured by estimating the color: brown staining indicated protein expression area, while shade represented the level of protein expression.

### 2.6 Western blot analysis

Samples of 50 mg were weighed, and the Cell lysis kit (Bio-Rad, Hercules, CA) was then employed as a means of extracting protein on ice with fresh protease and phosphatase inhibitors, as well as freshly added Phenylmethylsulfonyl fluoride. Following lysis and centrifugation, equivalent quantities of protein were run on 10% sodium dodecyl sulfate-polyacrylamide gel electrophoresis gels and transferred onto nitrocellulose membranes. Blocking buffer (Li-cor, Lincoln, NE) was used to block these membranes that were then stained using primary anti-IL-12p40 (1:500, Abcam), anti-MIP-1β (1:500, Abcam), anti-PDGF-BB (1:500, Abcam), anti-IL-1Ra (1:500, Abcam,), or anti-b-actin (1:2000, Santa Cruz Biotechnology, Dallas, TX) antibody for 12 hours at 4°C. A secondary antibody (Li-cor) diluted 1:10,000 was then used, followed by color detection via infrared laser imaging system (Odyssey, Li-cor).

### 2.7 Statistical analysis

Data are presented as mean ± standard deviation. One-way analysis of variance with the Least Significant Difference-*t* test was used for all comparisons, using the SPSS v. 22.0. The significance threshold was *P* < .05.

## 3 Results

### 3.1 Cytokine levels at diagnosis

The levels of following cytokines: soluble CD40 ligand, EGF, Eotaxin/CCL11, FGF-2, Flt-3 ligand, Fractalkine, G-CSF, granulocyte-macrophage colony-stimulating factor, GRO, IFN-α2, IFN-γ, IL-1α, IL-1β, IL-1Ra, IL-2, IL-3, IL-4, IL-5, IL-6, IL-7, IL-8, IL-9, IL-10, IL-12p40, IL-12p70, IL-13, IL-15, IL-17, IP-10, MCP-1, MCP-3, MDC (CCL22), MIP-1α, MIP-1β, PDGF-AA, PDGF-BB, RANTES, TGF-α, TNF-α, TNF-β, and vascular endothelial growth factor, were quantitatively tested. Among these cytokines, 4 cytokines, IL-12p40, MIP-1β, PDGF-BB, and IL-1Ra, showed significant differences in the B group relative to groups A and C group. Meanwhile, significant differences of the 4 cytokines are also shown in A group and C group (Table 1; *P* < .05 and *P* ≤ .001, respectively).

**Table 1:**
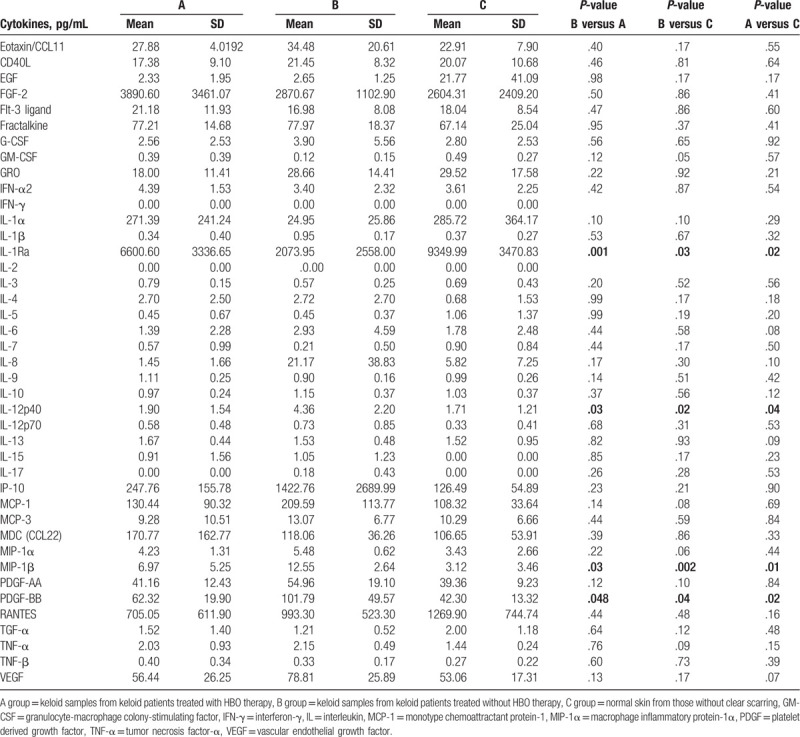
Cytokine levels (pg/mL) at diagnosis.

### 3.2 Histological analysis

**Figure 1:**
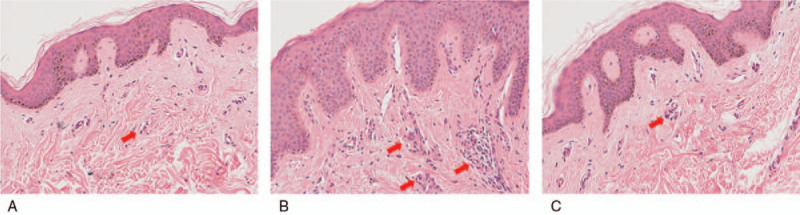
The results of epidermal and dermal H&E staining. The number of infiltrated cells (red arrow) was much lower in the A group and the C group than in the B group (images: 200×). A group = keloid samples from keloid patients treated with HBO therapy, B group = keloid samples from keloid patients treated without HBO therapy, C group = normal skin samples from patients without obvious scarring, H&E = hematoxylin and eosin.

H&E-stained sections allowed for the pathologic examination and to assess the inflammatory condition (Fig. 1). Different pathologic morphological structures were observed in the A, B, and C groups. Infiltrated inflammatory cells were barely observed in the A and C groups, with higher levels in the dermis of group B. As such, there was active inflammatory infiltration in keloid tissues treated without HBO therapy.

### 3.3 Immunohistochemical studies of IL-12p40, MIP-1β, PDGF-BB, and IL-1Ra

**Figure 2:**
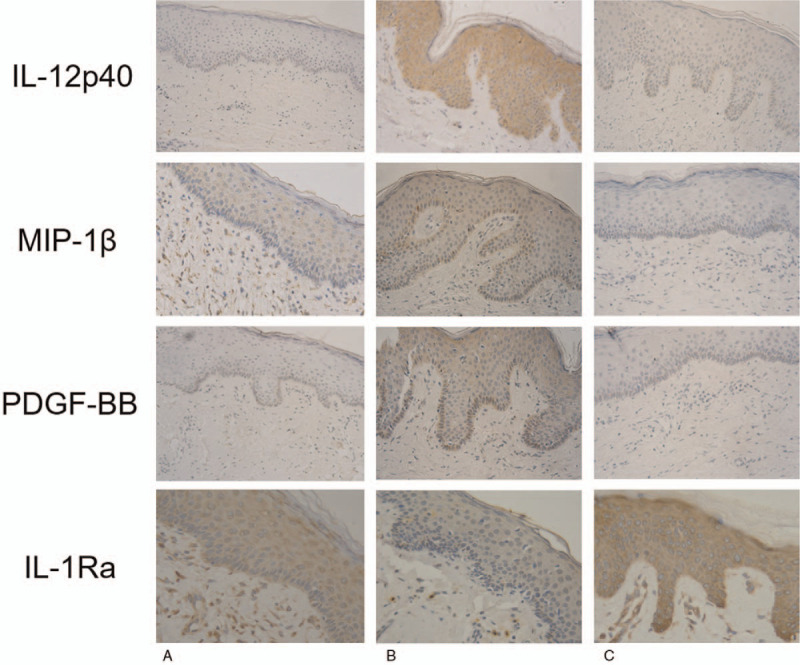
Representative micrographs (400×) of immunohistochemical studies for IL-12p40, MIP-1β, PDGF-BB, and IL-1Ra in all the 3 groups are shown. Brown indicates positivity, while how dark the brown is corresponds to the protein levels. The levels of IL-12p40, MIP-1β, PDGF-BB is overexpressed in group B relative to the A and C groups, while the IL-1Ra shows lower expression level in B group than in the A and C groups. Meanwhile, the levels of IL-12p40, MIP-1β, PDGF-BB is overexpressed in group A relative to the C group, while the IL-1Ra shows lower expression level in A group than in the C group. IL-12p40 = interleukin-12p40, IL-1Ra = interleukin-1 receptor antagonist, MIP-1β = macrophage inflammatory protein-1β, PDGF-BB = platelet-derived growth factor-BB.

We used immunohistochemistry to assess IL-12p40, MIP-1β, PDGF-BB, and IL-1Ra levels. The results revealed clearer IL-12p40, MIP-1β, and PDGF-BB levels in group B relative to A or C (Fig. 2), but the expression level of IL-1Ra was lower in the B group as compared with the other 2 groups. Meanwhile, the levels of IL-12p40, MIP-1β, PDGF-BB is overexpressed in group A relative to the C group, while the IL-1Ra shows lower expression level in A group than in the C group.

### 3.4 Expression of IL-12p40, MIP-1β, PDGF-BB, and IL-1Ra proteins

**Figure 3:**
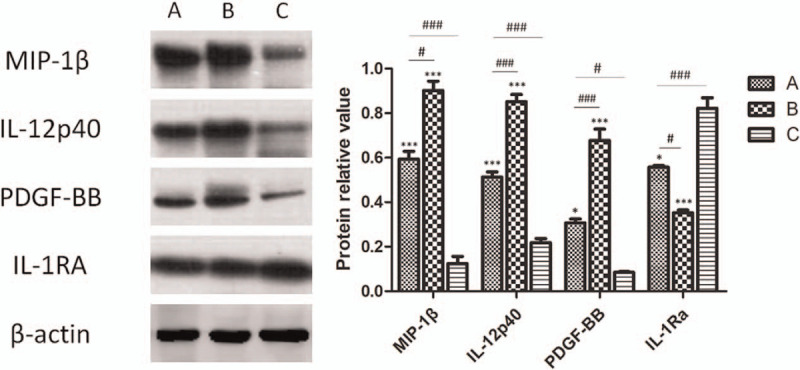
Protein expression of IL-12p40, MIP-1β, PDGF-BB, and IL-1Ra. Representative images of western blots for IL-12p40, MIP-1β, PDGF-BB, and IL-1Ra are shown. Densitometry analysis of IL-12p40, MIP-1β, PDGF-BB, and IL-1Ra protein levels is shown above, and the results are consistent with the immunohistochemistry studies. Values are expressed as means ± SD (n = 10, ^∗^*P* < .05 vs the C group, ^∗∗∗^*P* < .001 vs the C group, ^#^*P* < .05, ^###^*P* < .001). IL-12p40 = interleukin-12p40, IL-1Ra = interleukin-1 receptor antagonist, MIP-1β = macrophage inflammatory protein-1β, PDGF-BB = platelet-derived growth factor-BB, SD = standard deviation.

The protein expression of IL-12p40, MIP-1β, PDGF-BB, and IL-1Ra in samples of the 3 groups was visualized and analyzed by western blot (Fig. 3 and Table 2). The expression levels of IL-12p40, MIP-1β, and PDGF-BB were much greater in group B compared to the other 2 groups. But the expression level of IL-1Ra was much lower in the B group, with a significant difference compared with the A and C groups. Meanwhile, the levels of IL-12p40, MIP-1β, PDGF-BB is overexpressed in group A relative to the C group, while the IL-1Ra shows lower expression level in A group than in the C group, and the results are consistent with the immunohistochemistry studies.

**Table 2:**
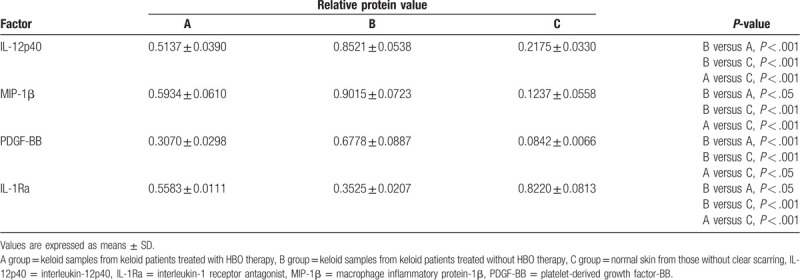
Relative protein value in all groups.

## 4 Discussion

Keloids are fibroproliferative tumors that occur due to excessive and abnormal accumulation of type I collagen in the dermis, in response to trauma or surgery, on the skin of susceptible individuals. Individuals of Asian, African, or Hispanic descent appear to be at a higher risk for developing keloids, which exerts a functional, aesthetic, or psychological effect on patients.^[7]^ The formation of keloids is a complex pathological process that is under the influence of various factors, including the surrounding environment, heredity, microenvironment, inflammatory responses, and immunity. Recent research has indicated that different types of human leukocyte antigens,^[8–11]^ several genes (*PTEN*^[12]^ and *P53*^[13]^), specific factors (chemokine-like factor-1,^[3]^ Smad interacting protein 1,^[14]^ insulin-like growth factor-1,^[15]^ and epithelial-to-mesenchymal transition) phenomenon^[16,17]^ are involved in the mechanism of keloid pathogenesis. Inflammatory mediators are key to keloid microenvironment, and closely related to hyperproliferation and abnormal expression of keloid fibroblasts. Overexpression of inflammatory factors in keloid tissue suggests that inflammatory responses are of crucial importance in the progression of keloid disease, and inflammatory responses are particularly pronounced in the reticular dermal layer.^[2]^

As small functional proteins, cytokines play an essential role in the inflammatory reaction and responses in keloid disease.^[18,19]^ According to the results of our study, IL-12p40, MIP-1β, PDGF-BB, and IL-1Ra might exert important and potential effects on inflammation and chemotactic activity. IL-1β is an important inflammatory and immune regulatory factor,^[20]^ and IL-1Ra is a competitive inhibitor of the IL-1 binding to IL-1R, and it specifically binds to IL-1R. IL-1Ra competitively inhibits IL-1β binding to IL-1R, and it exerts an inhibitory effect on the IL-1β-mediated inflammatory response.^[21,22]^ As a pro-inflammatory factor, IL-12 mainly has 2 subunits, p40 and p35. These 2 subunits are connected to each other through a disulfide bond, to form p70, which has relevance to immune regulation and inflammation.^[23,24]^ MIP-1β is 1 of the 4 subtypes of the MIP-1 CC chemokine subfamily, which are termed as CCL3 (MIP-1α), CCL4 (MIP-1β), CCL9/10 (MIP-1δ), and CCL15 (MIP-1γ).^[25]^ MIP-1β protein has chemotactic and pro-inflammatory effects but also drives homeostasis, which can activate the intracellular signal transduction pathways and stimulate the effector cells to secrete IL-6, TNF-α, IFN-γ, and other inflammatory factors to mediate the inflammatory responses and to regulate the synthesis of inflammatory factors.^[26,27]^ As an important inflammatory factor, PDGF tends to undergo dimerization by binding to the receptors and phosphorylation of its own tyrosine residues, and to promote the expression and activity of nuclear factor-кB and other inflammatory factors^[20,28]^; thereby activating the downstream signaling pathways to exert relational biological effects. However, the specific role of these cytokines in keloid development or progression is still not clear.

Various therapeutic strategies, including HBO therapy, have been used in clinical work, which shows a growing interest in new medical and surgical fields. HBO refers to exposure to 100% oxygen under elevated atmospheric pressure in a special treatment chamber, and it is recognized as a valuable supplementary therapeutic strategy in the field of plastic surgery. How HBO mediates protection is poorly understood, and as such we investigated its relation with the protection against inflammatory responses.

In our study, 3 groups were created and samples from 30 patients were analyzed to evaluate the effect of HBO therapy on the inflammation of keloid samples. The histological analysis by H&E staining showed more infiltrated cells in the keloid samples treated without HBO therapy, which indicates the presence of active inflammatory responses in keloid tissues. Higher protein expression levels of IL-12p40, MIP-1β, and PDGF-BB and lower IL-1Ra expression in the B group indicate that a higher degree of inflammation was present in keloid tissues than in the other 2 groups, and it showed more inflammatory cell infiltration. However, no significant difference was observed in IL-6, IL-8, or IL-10 expression, which is not consistent with the results of former studies,^[29,30]^ and it perhaps is related to the choice of this research sample.

According to the existing studies, the specific roles of IL-12p40, MIP-1β, PDGF-BB, and IL-1Ra in the pathogenesis, development or progression of keloid is still not clear. Rei Ogawa^[2]^ defined “keloid” as strongly inflamed pathological scars, which has close or caused association with the microenvironment of the keloid lesion. 15% of genes in keloid fibroblasts, the most important inflammatory factors, were upregulated, suggesting that keloid fibroblasts are crucial in the development and progression of inflammation.^[31]^ Inflammatory mediators, being an indispensable component in keloid microenvironment, play an essential role in the keloid microenvironment and are crucial for keloid fibroblast abnormalities.^[32]^ Therefore, we hypothesized that the proinflammatory genes in the keloid lesion area are stimulated by the inflammatory response in the microenvironment, leading to the pathogenesis of keloids. On the basis of these results in our study, we suggested that IL-12p40, MIP-1β, PDGF-BB, and IL-1Ra, the key cytokines with pro-inflammatory function, are the key players, and they may be considered as a common causative factor for keloid development.

Previous studies have shown that HBO therapy has been regarded as a successful adjunctive therapy for reducing inflammatory reactions^[33,34]^; however, how HBO therapy affects inflammatory responses in keloid tissue remains unknown. Postconditioning with HBO is commonly used to improve wound healing after flap transfer in plastic surgery.^[35]^ Recently, there has been research regarding the use of HBO as a therapy.^[36]^ Our study first assessed the expression levels of IL-12p40, MIP-1β, PDGF-BB, and IL-1Ra in keloid tissues in comparison with normal tissues, and it concluded that the expression of IL-12p40, MIP-1β, PDGF-BB, and IL-1Ra and inflammatory responses may be the causes of a keloid. In addition, the effects of HBO therapy on IL-12p40, MIP-1β, PDGF-BB, and IL-1Ra expression have also been assessed. The results showed that the level of inflammatory responses in keloid tissue was significantly decreased after 7 days of HBO therapy.

Interleukins, growth factors, and macrophage inflammatory proteins are common inflammatory or pro-inflammatory factors, and interleukins and growth factors play a paramount role in the initiation of inflammatory responses.^[37]^ Analyses of the expression levels of IL-12p40, MIP-1β, PDGF-BB, and IL-1Ra via immunohistochemical and western blot studies showed similar results. In the B group, higher expression levels of IL-12p40, MIP-1β, and PDGF-BB and a lower expression level of IL-1Ra were obviously observed. On basis of these results, HBO therapy effectively ameliorated the inflammatory condition of the keloid tissue. This effect may be caused by an increase in oxygen within the keloid tissue due to HBO therapy. This indicates that the oxygen-rich environment meets the need of high metabolism of a keloid, inhibits the secretion and release of inflammatory factors, and attenuates the level of the inflammatory response.^[38,39]^ HBO therapy significantly decreased the expression levels of IL-12p40, MIP-1β, and PDGF-BB, and it increased the expression level of IL-1Ra; thus, suggesting that HBO therapy has protective effects against the inflammatory responses during the progression of a keloid. This finding provides a new theoretical basis for ameliorating the microenvironment of keloid tissue.

This study mainly focused on the effect of HBO therapy on keloid tissue, thus, it did not clarify the mechanism of the pathogenesis and progression of keloid with inflammatory responses, the roles of IL-12p40, MIP-1β, PDGF-BB, and IL-1Ra, or the detailed way these factors work. Furthermore, the effect of HBO therapy on the inflammatory responses in patients of different ages also requires further study.

## 5 Conclusions

In this study, the HBO-treated keloid tissue demonstrated lower expression levels of IL-12p40, MIP-1β, and PDGF-BB and a higher expression level of IL-1Ra. These results indicate that HBO therapy can ameliorate the inflammatory levels of keloid tissue and that it has protective effects against the inflammatory responses.

## Acknowledgment

The authors would like to thank LetPub for providing linguistic assistance during the preparation of this manuscript.

## Author contributions

**Conceptualization:** Mingzi Zhang, Lin Zhu.

**Data curation:** Yan Hao.

**Formal analysis:** Yan Hao.

**Funding acquisition:** Youbin Wang.

**Investigation:** Hao Liu.

**Methodology:** Xinhang Dong.

**Project administration:** Youbin Wang.

**Software:** Mingzi Zhang.

**Supervision:** Lin Zhu.

**Writing – original draft:** Xinhang Dong.

**Writing – review and editing:** Youbin Wang.
